# In silico generation of gene expression profiles using diffusion models

**DOI:** 10.1186/s12859-026-06470-8

**Published:** 2026-05-31

**Authors:** Alice Lacan, Romain André, Michèle Sebag, Blaise Hanczar

**Affiliations:** 1 IBISC, University Paris-Saclay (Univ. Evry), Évry-Courcouronnes, France; 2 TAU, LISN-Inria-CNRS, University Paris-Saclay, Gif-sur-Yvette, France; 3 Diagnostic Image Analysis Group, Department of Medical Imaging, Radboud University Medical Center, Nijmegen, Netherlands

**Keywords:** Transcriptomics, Precision medicine, Deep generative models, Diffusion

## Abstract

**Background:**

RNA-seq data is used for precision medicine (e.g., cancer predictions), which benefits from deep learning approaches to analyze complex gene expression data. However, transcriptomics datasets often have few samples compared to deep learning standards. Synthetic data generation is thus being explored to address this data scarcity. So far, only deep generative models such as Variational Autoencoders (VAEs) and Generative Adversarial Networks (GANs) have been used for this aim. Considering the recent success of diffusion models (DM) in image generation, we propose a diffusion-model-based generation pipeline that leverages the power of such generative models on transcriptomics data.

**Results:**

This paper presents two state-of-the-art diffusion models (DDPM and DDIM) and achieves their adaptation in the transcriptomics field. DM-generated data of L1000 landmark genes show better predictive performance over TCGA and GTEx datasets. We also compare linear and nonlinear reconstruction methods to recover the complete transcriptome. Results show that such reconstruction methods can boost the performance of diffusion models, as well as VAEs and GANs.

**Conclusions:**

Overall, the extensive comparison of various generative models using data quality indicators shows that diffusion models rank among the best-performing methods, making them promising synthetic transcriptomics generators.

## Introduction

Precision medicine is advancing rapidly by leveraging multimodal patient data, ranging from medical imaging and electronic health records (EHRs) to genomics. The potential of each data source is investigated extensively, using deep learning in computer vision or natural language processing (NLP) for, respectively, medical imaging and EHRs, yet more modestly in genomics. The genomics domain, encompassing a variety of high-dimensional data like transcriptomics, holds valuable information regarding the prediction of patients’ diagnoses, prognoses, and treatment responses. Next-generation sequencing (NGS) methods [1] make it possible to afford significant RNA sequencing (RNA-seq) data at a lower cost and high accuracy. This data surge is set to fundamentally impact precision medicine to develop person-dependent treatments. For instance, bulk RNA is increasingly used to predict tissue-specific conditions, cancer diagnoses, and prognoses [2–4].

On the methodological side, advanced deep learning methods awaken great hopes as they enable representing and learning complex non-linear relationships from high-dimensional data [5]. In practice, however, the current scarcity of gene expression data [6] remains a crippling problem for the use of deep learning. Deep models notoriously require massive amounts of training data, all the more so in high-dimensional spaces (up to 22,000 coding genes). In transcriptomics, the number of samples is restricted due to the limited number of patients, residual costs of RNA sequencing methods, and the need for cohort integration.

**Fig. 1:**
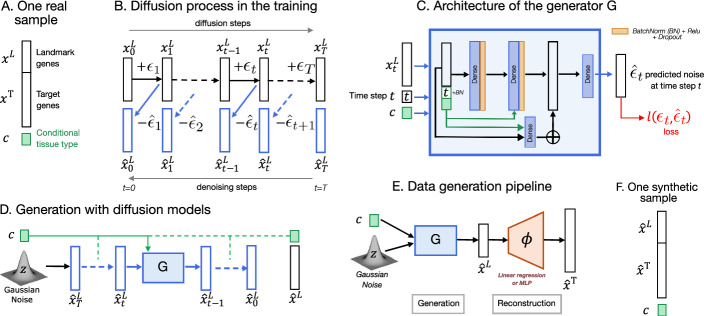
Overview of our data generation pipeline with a diffusion model (DM). For each real sample **A** we consider the L1000 landmark genes $x^L$ and the remaining target genes $x^T$, as well as a corresponding covariate *c* (tissue type). During the training of the DM (**B**), the model **C** learns to progressively denoise the samples for which Gaussian noise $\epsilon _t$ was added at each time step *t*. The model takes as input a noisy sample $x_t^L$ and directly predicts this added noise. It is conditioned on both the time step *t* and the tissue type *c*. For the generation process (**D**), the DM takes as input Gaussian noise and progressively denoises the sample, exactly like in training (**B**). The overall pipeline **E** consists of a generation phase, followed by a reconstruction phase taking as input the generated landmark genes $\hat{x}^L$ to recover the final synthetic sample with target genes $\hat{x}^T$ (**F**)

This scarcity makes for insufficiently representative datasets, resulting in a poor generalization, that is, overfitting. Building efficient deep neural models from small datasets thus often relies on domain knowledge and computationally intensive efforts (e.g., to regularize the learning criterion and adjust the method hyperparameters).

The automatic generation of sizeable synthetic transcriptomics datasets, faithful to the data distribution, is a sound way to address the shortage of data. The generation of synthetic samples can also enable the target tasks to be conducted on anonymized synthetic data, thus addressing privacy concerns [7]. In computer vision and NLP, *data augmentation* is a regularization technique exploiting additional artificial training samples that follow the same data distribution as the original training set. Deep generative models like Variational Auto-Encoders (VAEs [8]) and Generative Adversarial Networks (GANs [9]) have been presented as good candidates for generating such synthetic samples [10].

For cancer applications, a promising WGAN-GP data generation strategy was proposed by [11]; the classifier trained on generated samples yielded state-of-the-art prediction results on bulk RNA data. [12–14] presented GAN-based data augmentation approaches to boost performances using conditional GANs and better evaluate data quality. Interestingly enough, the latent representation of VAEs and GANs were also leveraged to capture biologically relevant features for cancer-related tasks [15] and interpretations of biological pathways [16]. However, [14] showed that synthetic data diversity and mode collapse remained challenging for such generators.

More recently, a new type of generative model referred to as diffusion models (DMs) [17] are revolutionizing image generation with, for example, DALL-E 2 [18]. Diffusion models are celebrated for their ability to generate a large diversity of high-quality samples and offer new interpolation possibilities.

While diffusion has been successfully applied to protein generation with FoldingDiff [19], its application in transcriptomics is in its infancy. [20] recently proposed scDiffusion, a latent diffusion model which involves multiple classifiers to guide the diffusion process and an interpolation strategy that allows the generation of cell development trajectories from a given cell state. Results are promising but the authors benefited from the well-structured latent space of an autoencoder pre-trained over 22.7 million single-cell samples. Applying such methods to limited bulk RNA datasets is less clear. Other recent work by [21] leveraged diffusion with graph networks for drug response prediction. Diffusion models have not yet been efficiently applied to bulk transcriptomics to our best knowledge (except for [22], reporting an unsuccessful application). A primary challenge lies in the scarcity of transcriptomics data of high dimensionality and complex structure.

Latent diffusion approaches run diffusion in a learned representation space and have shown success in reducing effective dimensionality of images while improving generated quality [23]. Related ideas have been less explored for other high-dimensional modalities such as tabular data since they require very large and carefully crafted models to balance reconstruction and generation tasks [24]. State-of-the-art diffusion models for tabular data such as TabDDPM [25], TabSyn [26] and TabDiff [27] have demonstrated great capacities on large *n* (*>10,000*), small *p* (*<50*) scenarios only.

Building upon the methodology used to deploy and assess data augmentation with VAEs and GANs [14], the proposed contribution is a gene expression generation pipeline leveraging the power of diffusion models. The critical issues related to the high dimensionality of transcriptomics data are handled by considering the so-called 1,000 landmark genes [28]: the data generation is conducted in this reduced 1,000-dimension space, and the generated samples are mapped to the remaining genes space using a trained linear or non-linear approach [29, 30]. To the best of our knowledge, this is the first successful demonstration of competitive diffusion models on bulk RNA-seq data.

## Methods

**Fig. 2:**
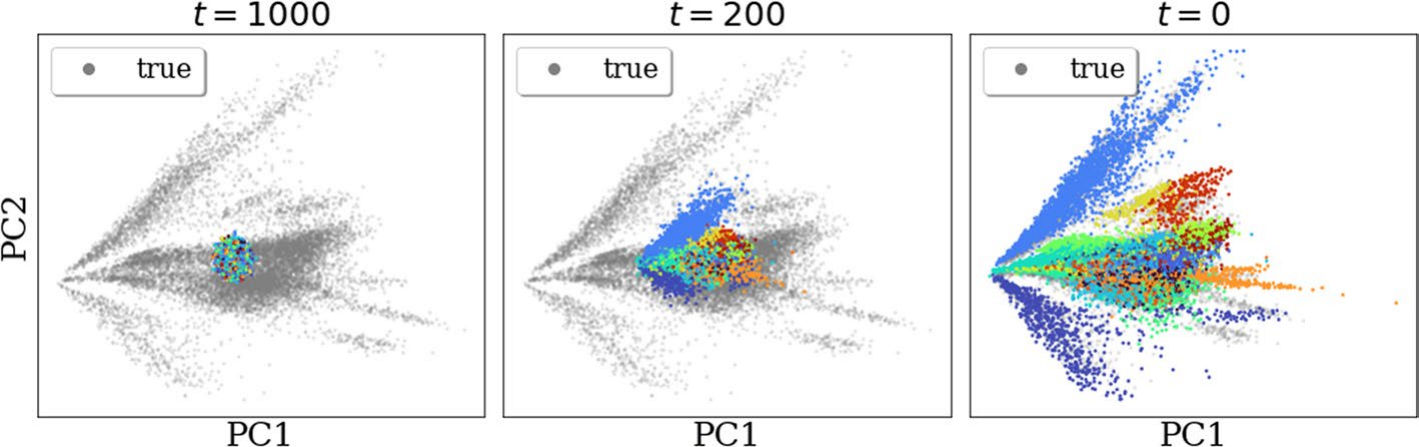
PCA visualization of the 1st and 2nd principal components throughout the generation steps *t = 1000,..., 0* for GTEx data (L1000) and the DDIM-generated samples $x_t$. Colors highlight the different tissue clusters

This section presents the core contribution of the paper, the diffusion-model-based data generation methodology illustrated in Fig. 1. For the sake of conciseness, the formal background of DMs is first introduced and discussed, referring the reader to the original papers for a more comprehensive presentation, and to Appendix A for details on the baseline VAEs and GANs. An original methodology is also proposed to address the high dimensionality issue inherent in transcriptomics data, which constitutes a significant hurdle for DM-based generative models.

Notations: In the following, $\mathcal {D}_{t}$ denotes the (unknown) true data distribution, and $\mathcal {D}_g$ the learned distribution parameterized from the model parameters *θ *. Deep generative models proceed by learning a distribution $\mathcal {D}_g$= $p_{\theta }(x)$ that approximates the target distribution $\mathcal {D}_{t}$, supporting a sampling mechanism.

### Diffusion-based generation method

Early success in stable diffusion-based generative modeling is due to [17]. However, widespread interest arose with the introduction of Denoising Diffusion Probabilistic Models (DDPMs) by [31] and [32], outperforming GANs in terms of class and mode coverage, image quality, and stability, making them prominent generative models.

Denoising diffusion probabilistic models: [31] introduce a Markov Chain framework called diffusion. In forward mode, starting from an initial example $x_0 \sim \mathcal {D}_{t}$, a sequence of *T* samples $x_1, \ldots x_T$ is simulated, where $x_t$ is obtained by adding Gaussian noise to $x_{t-1}$ (Fig. 1, panel B):

1 $$\begin{aligned} q(x_t | x_{t--1}) = \mathcal {N}\left( x_t; \sqrt{1 -- \beta _t}x_{t--1}, \beta _tI\right) \end{aligned}$$

The noise level $\beta _t \in [0,1]$ gradually increases along a noise schedule, ensuring that the final $x_T$ is essentially a pure Gaussian noise sample. A property of the DM forward process is that the intermediate $x_t$ are in the same input space as $x_0$: the forward process only depends on the (fixed) noise schedule $\beta _t$.

Besides the iterative process (Eq. 1), the forward process can also be computed in closed form as in Eq. 2, enabling the direct modelling of all noisy $x_t$ from the initial sample $x_0$ and Gaussian noise $\epsilon \sim \mathcal {N}(0, I_d)$.

2 $$\begin{aligned} x_t = \sqrt{\bar{\alpha _t}}x_0 + \sqrt{1 -- \bar{\alpha _t}}\epsilon \text {where} \alpha _t = 1 -- \beta _t \text {and} \bar{\alpha _t} = \Pi _{t}^{s=0} \alpha _s. \end{aligned}$$

The closed-form computation speeds up training compared to the iterative computation; it promotes a stable setting and alleviates memory storage issues.

The generative model corresponds to the backward process trained to invert the forward process, achieving the progressive denoising of each sample $x_T$ (Fig. 2, left to right). Formally, the generator learns the reverse process defined as:

$$\begin{aligned} \mathcal {D}_g= p_{\theta }(x_{t-1}| x_t) \end{aligned}$$

where *θ * denotes the parameters of the neural network optimized to estimate the loss function (below). In the original computer vision applications, the prevalent denoiser backbone is typically a U-NET architecture [33], which is a symmetric encoder–decoder with skip connections between corresponding resolution levels obtained by down/up sampling images.

The loss function used to train the model is the KL divergence between the joint distributions of the forward and reverse Markov chains. In addition, the parameterization is simplified to yield a more smooth loss function. Along this line, the generative sampling produces a denoised sample $x_{t-1}$ from $x_t$ and $\epsilon _{\theta }$:

3 $$\begin{aligned} x_{t-1} = \frac{1}{\sqrt{\alpha _t}}(x_t - \frac{\beta _t}{\sqrt{1 - \bar{\alpha _t}}}\epsilon _{\theta }(x_t, t)) + \sigma _tz \text { with} z \sim \mathcal {N}(0, I_d)\end{aligned}$$

where $\epsilon _{\theta }$ is the output of the trained neural network, computed from $x_t$ and *t*. Simply put, the DM generative model is trained to directly predict the noise added on sample $x_t$ at time step *t* during the forward process, of the same dimension as $x_t$ (Fig. 1, panel C).

Eventually, the simplified loss function is the mean squared error:

$$\begin{aligned} L_{\text {simple}}(\theta ) = \mathbb {E}_{x_0, \epsilon }\Vert \epsilon - \epsilon _{\theta }(x_t, t)\Vert _2^2 \end{aligned}$$

where $x_t$ is computed from Eq. 2. The smoothness of the loss function leads to significantly more stable training compared to, e.g., the GAN min-max loss.

Despite the success of DDPMs, a notable challenge lies in the inference time required for generating samples. A generated sample is produced by: i) sampling $x_T$ from the prior $\mathcal {N}(0, I_d)$; ii) *T* times, iteratively sampling $x_{t-1}$ using $x_t$ and $\epsilon _{\theta }(x_t, t))$. This process implies *T* passes through the neural network (as opposed to one pass in a VAE or a GAN). As many diffusion steps (*T ≈ 1,000*) are recommended to generate high-quality samples, time inference remains a major bottleneck for the practicality of such diffusion models.

Denoising diffusion implicit models: [34] introduce Denoising Diffusion Implicit Models (DDIMs) to enforce a good trade-off between the quality of the generated sample and the computational cost by redefining the diffusion process as a non-Markovian process. The DDIM framework is based on the same objective function and backbone neural network as the DDPM one: it can be applied post-training on top of a previously trained DDPM model.

It proceeds by replacing the variance term $\beta _t$ with $\sigma _t(\eta , \beta _t)$ where *η * is a positive value that controls the stochasticity of the generation process: with the same notations as in Eq. 2,

4 $$\begin{aligned} \sigma _t(\eta , \beta _t) = \eta \sqrt{\frac{1- \bar{\alpha }_{t-1}}{1- \bar{\alpha }_t}} \sqrt{1 - \frac{\bar{\alpha }_{t}}{\bar{\alpha }_{t-1}}} \end{aligned}$$

In the forward process (Eq. 1), $x_t$ now depends on both $x_{t-1}$ and the initial sample $x_0$, making the forward process non-Markovian, though it preserves the closed-form property of the DDPM (Eq. 2). With the use of $\sigma _t(\eta , \beta _t)$, the stochasticity of the forward process can be controlled. For *η =1*, one falls back on the DDPM setting. For *η = 0*, the forward process is deterministic in the sense that $x_{t-1}$ can be directly computed from $x_0$ and $x_t$. In such a case, the backward process is also (trained to be) deterministic: from a given input noise $x_T$, the same generated sample $x_0$ is always derived. This deterministic process entails nice properties, such as the ability to interpolate among generated samples by applying the backward process to the interpolation of their input noise samples.

Overall, the denoising step in Eq. 3 becomes:

5 $$\begin{aligned} x_{t-1} = \sqrt{\bar{\alpha }_{t-1}}(\frac{ x_t - \sqrt{1 - \bar{\alpha _{t}}}\epsilon _{\theta }(x_t, t) }{\sqrt{\bar{\alpha _{t}}}} ) + \hat{x_0} + \sigma _tz \end{aligned}$$

with $$\hat{x_0} = \sqrt{1 - \bar{\alpha _{t-1}} - \sigma _t^2}\epsilon _{\theta }(x_t, t)$$ and $$z \sim \mathcal {N}(0, I_d)$$.

The same loss function $L_{\text {simple}}$ is used to optimize the model. The difference is that the denoising pass (Eq. 5) enables to skip intermediary steps to increase generation speed. When the process is deterministic, the resulting generated samples have the same high-level features and only differ in details, depending on the number of skipped steps.

According to the authors, DDIMs outperform DDPMs when the number of diffusion steps is lower than the initial *T* steps. The transition from DDPMs to DDIMs holds promise for addressing scalability challenges and extending the applicability of generative models across diverse domains.

### Adapting DDIM to the high dimensional transcriptomics space

The proposed approach builds upon [28], which investigated leveraging redundancy and correlations in gene expression data to enhance sequencing efficiency and resource allocation. The authors introduce an ensemble comprising approximately 1,000 landmark genes, referred to as the L1000 genes; they employ linear regression to infer the expression data of all other genes (19,000) from the L1000 genes.

Subsequently, [29] and [30] proposed neural learning approaches using the dimensionality reduction permitted by the L1000 genes to achieve gene expression inference via multilayer perceptrons (MLPs) architectures. Operating in the reduced L1000 space is shown to efficiently address the instability and memory consumption problems that can be encountered when training large deep generative models on such high-dimensional data.

The presented study proceeds by conducting the data generation phase in the L1000 landmark genes space noted $L_g$. The generated samples are mapped to the remaining target genes space noted $T_g$, using either a trained linear regression (LR) or a multilayer perceptron (MLP) method. Both methods are trained by minimizing the Mean Squared Error (MSE) between the true target genes and reconstructed genes (Eq. 6) on the validation set. The Mean Absolute Error (MAE) is additionally employed to facilitate a comprehensive comparison of the reconstruction performances, mitigating the influence of outliers. We refer to the complete transcriptome as the ensemble $L_g \cup T_g$.

6 $$\begin{aligned} L_{recon} = \underset{y \in T_g}{\mathbb {E}}||y - \hat{y}||_2^2 \end{aligned}$$

with $x \in L_g$ the input in reduced dimension space, $y \in T_g$ its representation in the target genes space, and $\hat{y} = \phi (x)$ its approximation by the reconstruction model *ϕ *. This reconstruction model is then used to map the synthetic landmark genes onto the target genes (Fig. 1, panel E).

### Baselines

We compare diffusion models with two strong generative baselines: a variational autoencoder (VAE) [8] and a Wasserstein GAN with gradient penalty (WGAN-GP) [35]. The VAE learns a regularized latent representation and decodes from this space to generate diverse samples. The WGAN-GP trains a generator and critic adversarially to minimize an estimate of the Wasserstein-1 distance, which often yields high-fidelity samples. All models are trained on the same input data and conditioned on the same covariates used in this study (tissue type). Architectural details and hyperparameters are provided in Appendices A and C.

## Experimental setting

This section describes the experimental settings for reliable model comparisons between a VAE, a WGAN-GP (Appendix A), and our DDIM. All final models are trained on a single NVIDIA A40 GPU with 48 GB of RAM. Reported standard deviations reflect variability across five independent training runs (random seeds and training stochasticity), and are therefore interpreted as a measure of training stability.

### Benchmark RNA-seq datasets

The presented experiments consider the Pan-Cancer Genome Atlas (TCGA) [36] and the Genotype-Tissue Expression (GTEx) [37]. TCGA data was retrieved using the RTCGA^1^ package in R (release date 2016–01-28), while the latest version (v8) of GTEx data (with TPM normalization) was obtained from the open-access portal.^2^ The preprocessing procedure, involving removal of duplicates, landmark and target genes IDs mapping, and standardization, can be found in our open-source code.^3^ The final TCGA dataset includes 6,499–1,625–1,625 train-validation-test samples of 978 landmark genes and 19,553 target genes to reconstruct. The GTEx dataset contains 9,796–2,448–5,000 train-validation-test samples of 974 landmark genes and 17,717 target genes. We consider 24 tissue types for TCGA and 26 for GTEx.

### Data quality evaluation

Our data quality evaluation methodology follows the guidelines in [14], with unsupervised and supervised indicators presented below (see Appendix B for detailed mathematical definitions).

- Reverse Validation accuracy assesses the predictive capacity of generated data using an MLP trained on generated data and tested on the true test data unseen during training.
- Fréchet Distance (FD) [38] measures the similarity between true and generated data based on the last hidden layer of an MLP trained to discriminate tissue types, accounting for similarity in a meaningful reduced space.
- Precision and Recall (PR) [39] measure the distance between true and fake distributions considering the intrinsic dimensionality of their support, computed by nearest-neighbor-based manifold approximation.
- F1-score represents the harmonic mean between previously mentioned precision and recall measures. There is no direct interpretation of this metric except as a compact trade-off summary between precision and recall.
- Adversarial Accuracy (AA) [7], assesses whether generated data balance accuracy and privacy in the context of sensitive data, with an optimal value of 0.5.
- Correlation Score [11] compares the correlation matrices of true and generated data using Pearson correlation scores.

**Unsupervised Indicators**

Notably, [38] and [39] highlight the sensitivity of FD and PR metrics to the number of considered samples, prompting compliance with the computation methodology from [14]. These indicators were used to assess the quality of both the generated data and the reconstructed data, in addition to the MAE. A qualitative assessment was also performed using dimensionality reduction visualizations such as Uniform Manifold Approximation and Projection (UMAP) and Principal Components Analysis (PCA). Finally, we report adversarial accuracy as a proxy for distinguishability: values near 0.5 indicate near-chance separation for a classifier, whereas higher values indicate that synthetic samples are more easily distinguished from real data under this adversary.

Supervised Indicators: The baseline performance indicator based on the predictive accuracy is computed by training an MLP, optimized through a Bayesian optimization [40] over 1,000 trials on the validation sets. We considered the multiclass classification task of the L1000 landmarks genes and the ensemble $L_g \cup T_g$) over tissue types. The reverse validation is based on the same best fixed MLP architecture trained on the generated data only.

Interpreting indicators: We evaluate synthetic gene expression using complementary indicators that capture different failure modes: (i) fidelity (how realistic generated samples are), (ii) diversity/coverage (whether the generator reproduces the variety of real samples), and (iii) downstream utility (whether models trained on synthetic data generalize to real data). In particular, Fréchet Distance (FD) summarizes distributional similarity between real and synthetic samples in an embedding space: smaller FD indicates closer match (FD = 0 only when the embedded distributions match in mean and covariance). Precision/Recall (PR) explicitly separates fidelity (precision) from coverage (recall): higher precision means generated samples lie in regions supported by real data, while higher recall means the generator covers the support of the real distribution. We include additional measures such as adversarial accuracy and gene–gene correlation agreement to probe separability and dependency structure.

### Model configuration

Deep generative models require a carefully selected training procedure due to their hyperparameter sensitivity. A set of best hyperparameters was selected for each generative model after maximizing the unsupervised F1 score (Section Data Quality Evaluation) through a Bayesian search of 100 trials. Embeddings of the tissue type covariates are used to condition all the generative models [11]. Details of the VAE and WGAN-GP baseline models optimization can be found in Appendix C, alongside the investigated hyperparameters.

The DDIM is trained with many epochs (15,000) due to numerous diffusion steps (1,000) and a more expressive architecture. We adapted the architecture as a residual block of the same input and output size (Fig. 1, panel C) because we could not use the typical U-NET model, unsuited to the characteristics of our tabular data. DMs also leverage the power of attention mechanisms and sophisticated class conditioning (e.g., classifier guidance in [41]), which we did not implement in this first adaptation. We used Automatic Mixed Precision [42] alongside a learning rate warmup strategy and big batch sizes (2,048) to keep an efficient training time. In addition to the residual block layers dimensions and the learning rate, we optimized the dropout rate, the variance ($\beta _t$) scheduler (constant, linear, or quadratic), and the conditioning time steps (with or without sinusoidal embedding). Input data scaling was also investigated, and scalings alternative to *[-1,1]* appeared to degrade training stability and performance. As stated by [34], the neural network reverse process needs to operate on consistently scaled inputs. A mismatch between signal magnitude and the noise schedule may lead to insufficient noising or sensitivity to numerical noise which motivated us to remain attentive to scaling. The training time is 1 h and 3 min for the DDIM on TCGA and 3 h and 7 min for GTEx. The best retained hyperparameters can be found in Appendix C.

### Ablation analyses

In the following result section, we also provide ablation studies for: (i) the diffusion sampling variant (DDPM vs DDIM via *η *), (ii) the number of inference steps (Table 3; Appendix 9), (iii) the reconstruction strategy (LR vs MLP; Appendix 10–12), and (iv) generating in reduced space (L1000) with reconstruction vs generating directly in the full space (Appendix 15). Together, these experiments characterize the main design choices of the proposed pipeline.

## Results

This section discusses the results obtained with the compared generative models: DMs, VAEs, and GANs, along with the performance indicators presented in Section Data Quality Evaluation.

**Table 1:** Comparing the unsupervised indicators performance of the different generative models on GTEx landmark genes

Metric	VAE	WGAN-GP	DDIM
Corr. (%) *↑ *	98.14 ± 0.08	**99.43 ± 0.04**	98.93 ± 0.33
Prec. (%) *↑ *	**99.44 ± 0.06**	99.26 ± 0.1	98.88 ± 0.05
Recall (%) *↑ *	59.28 ± 0.21	**90.47 ± 0.09**	89.23 ± 0.28
F1 (%) *↑ *	74.28 ± 0.1	**94.66 ± 0.1**	93.81 ± 0.09
AA (%)	**50.33 ± 0.45**	60.47 ± 0.27	46.47 ± 0.61
FD *↓ *	4.0768 ± 0.1212	**0.935 ± 0.0414**	3.8931 ± 1.9

Because FD/PR are sensitive to sample size, they are computed on the training split for stability, following the protocol in Appendix B. Best results are indicated in bold. *↑ *: the higher, the better. *↓ *: the lower, the better

**Table 2:** Comparing the unsupervised indicators performance of the different generative models on TCGA landmark genes

Metric	VAE	WGAN-GP	DDIM
Corr. (%) *↑ *	98.26 ± 0.04	98.45 ± 0.09	**99.18 ± 0.02**
Prec. (%) *↑ *	99.76 ± 0.03	95.28 ± 0.14	**99.99 ± 0.01**
Recall (%) *↑ *	70.13 ± 0.58	**91.16 ± 0.23**	71.98 ± 0.35
F1 (%) *↑ *	82.36 ± 0.06	**93.17 ± 0.17**	83.7 ± 0.02
AA (%)	**47.19 ± 0.54**	69.04 ± 0.53	44.54 ± 0.55
FD *↓ *	0.6269 ±0.0291	2.5603 ± 0.363	**0.5181 ± 0.0339**

Because FD/PR are sensitive to sample size, they are computed on the training split for stability, following the protocol in Appendix B. Best results are indicated in bold. *↑ *: the higher, the better. *↓ *: the lower, the better

### Data generation in the reduced L1000 space

In Fig. 2, the GTEx DDIM-generated data are represented in the 2D plane defined from the PCA analysis at each diffusion step, where the colors correspond to the different tissues. The diffusion trajectories over the 1,000 diffusion steps show that synthetic data are initially grouped in the center of the image (*t=1,000*) and gradually spread across true data points (in grey) when *t* goes to 0, nicely grouping together the tissue-related clusters. Similar nicely defined clusters can be found in the TCGA UMAP (Appendix D).

The quantitative analysis is presented in Tables 1 and 2, reporting the unsupervised performance indicators on GTEx and TCGA, respectively. On GTEx, DDIM consistently ranks second (except in precision), while VAE or WGAN-GP ranks first, depending on the indicator. All the models exhibit strong correlations and precision, exceeding at least 98%, indicating that the generated data closely resemble true data. Regarding diversity (true clusters are all visited by generated data), VAE is substantially outperformed by WGAN-GP and DDIM (from approximately 59% to 90%). VAE is also outperformed regarding the Fréchet distance.

In contrast, DDIM is better at generating TCGA data with high fidelity as the model ranks first regarding correlation, precision, and Fréchet distance. However, looking at recall, WGAN-GP substantially outperforms the other models that fail to reach more than 71% of diversity. A tentative interpretation is that both VAE and DDIM fail to capture sufficiently the heterogeneity of cancerous samples.

Regarding privacy, the adversarial accuracy (AA) is relatively stable around *∼ *0.5 for all models, except for WGAN-GP (0.6047 for GTEx, 0.6904 for TCGA), suggesting that it tends to generate data more easily distinguishable from the true dataset. The DDIM is second best after the VAE on both datasets.

**Table 3:** Comparative results in terms of recall (diversity) and the number of diffusion steps used in the generative process (the computational cost varies linearly with the number of diffusion steps)

	GTEx			TCGA		
Steps	50	100	1000	50	100	1000
DDPM (*η =1*)	0.82	0.86	**0**.**90**	0.64	**0**.**70**	**0**.**77**
DDIM (*η =0*)	**0**.**77**	**0**.**86**	0.89	**0**.**65**	0.68	0.72
Inference time (s)	14	28	268	6	14	127

Better results are indicated in bold

**Fig. 3:**
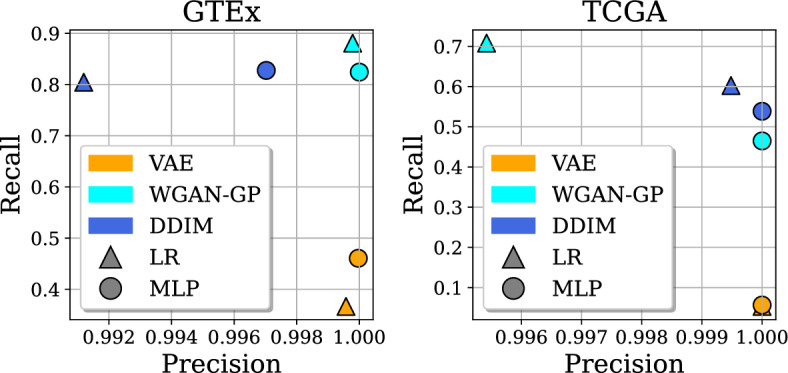
Precision/Recall trade-off according to the generative model, the reconstruction method and the dataset. Metrics are computed on the final sets of generated and reconstructed target genes ($L_g \cup T_g$)

The relationship between diversity and inference steps is shown in Table 3, comparing DDIM and DDPM (same trained model with *η =1*) and for a number *T* of diffusion steps $\{50, 100, 1000\}$ in the generation pass. The highest diversity is obtained with a DDPM using 1,000 diffusion steps for GTEx and TCGA; this result is consistent with a higher *η * leading to higher stochasticity in the generation process. In addition, a DDIM with 50 time steps yields a higher diversity (86.09%) than a DDPM with 100 steps (85.74%), thus dividing by half the inference time (from 28 sec. to 14 sec.). On TCGA, a DDPM with 100 steps reaches almost the same diversity (70.34%) as the best DDIM on 1,000 steps (71.70%), thus dividing by nine the inference time (from 127 sec. to 14 sec.). Skipping time steps in a DDIM framework can, therefore, efficiently decrease the generation cost while preserving good diversity (more in Appendix 9).

Regarding computational resources, diffusion models require larger neural architectures than VAEs and WGANs-GP (Appendix F). Reaching the same realism-diversity performance on GTEx requires a DDIM architecture bigger by an order of magnitude than for WGAN-GP (228 million parameters vs 19 million). On TCGA, the best DDIM is almost three times bigger than the best WGAN-GP. The VAE, which hardly reaches the same performances as WGANs-GP or DDIMs, might also need a bigger neural architecture.

In counterpart to their being more computationally demanding, DMs are easier to train and deliver more stable results than VAEs or WGANs-GP. In our experiments on TCGA, for instance, at least 18% of VAE runs (15% of WGAN-GP runs) fail to converge.

**Fig. 4:**
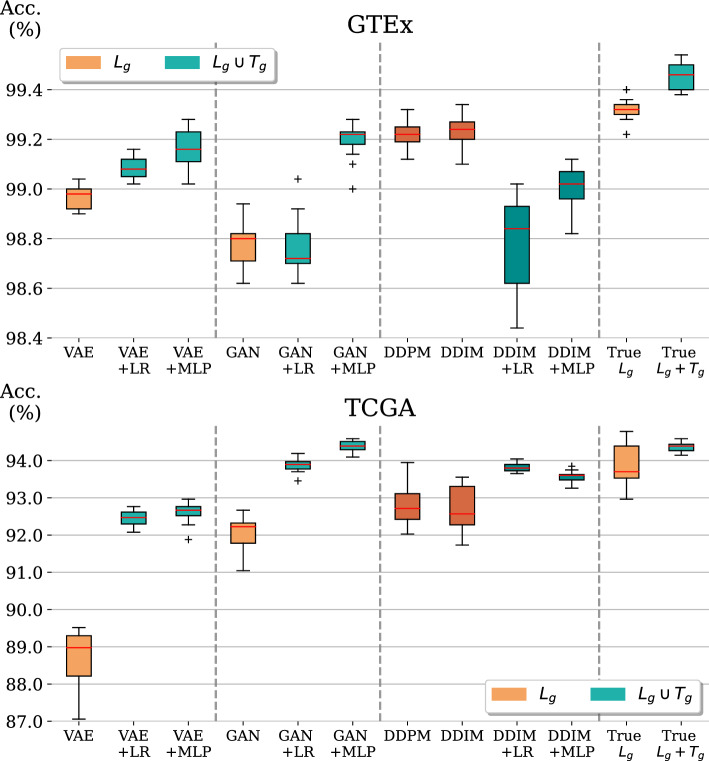
Predictive accuracy on tissue classification (supervised performance): on GTEx (top) and TCGA (bottom), compared with the baseline trained on the true data (rightmost). We differentiate between classification in reduced space $L_g$ (orange), full gene space $L_g \cup T_g$ (blue) and DM-generated data in darker colors. All classifiers associated with a given dataset are trained on the same number of samples, share the same architecture

### Generation of complete gene expression profiles

In Appendices 10-12, we first evaluated the linear regression and MLP reconstruction strategies using real landmark genes as inputs. Although the MLP achieves lower MAE (Appendix 10), both mappings yield comparable distributional quality under our unsupervised indicators. As a nonlinear mapper can reduce average error by smoothing extreme target-gene values, it may increase fidelity (precision) while slightly reducing diversity (recall), consistent with the recall decrease observed on TCGA (Appendix 12). Since MAE may underweight higher-order distributional properties (e.g., tail behavior), we report complementary indicators (PR/FD/correlation) on reconstructed data and retain both reconstruction approaches.

Regarding the unsupervised indicators, each pipeline’s PR performances are reported in Fig. 3. On average, the MLP mapping yields good precision, sometimes at the expense of a small loss in recall compared to LR. On GTEx (Fig. 3, left), the best option is the WGAN-GP + mapping (similar to the performance obtained on L1000). On TCGA (Fig. 3, right), DDIM catches up with WGAN-GP, and both are on the precision-recall Pareto front. As an upper-bound reference, Appendices 11–12 report performance when LR/MLP reconstruct from real landmark genes: the MLP exceeds 98% in both precision and recall on GTEx, and LR exceeds 95% on TCGA.

When observing the reverse validation accuracy in Fig. 4, the main finding is that DM-generated L1000 data (DDPM and DDIM) is closest to reaching baseline classification performances (with true data) on both datasets. This shows DM-generated data can be used directly in the reduced landmark gene space as an alternative synthetic dataset.

Interestingly, all methods’ performances are generally improved when mapping the generated data to the entire gene space (except DDIM on GTEx, displaying a minimal gap in order of magnitude). For WGAN-GP, the predictive accuracy is better when considering the data generation in L1000, followed by a mapping on $T_g$, compared to the data generation in $L_g \cup T_g$ directly (Appendix 15). For VAE, we observe a similar accuracy pattern on GTEx. On a more heterogeneous dataset like TCGA, the WGAN-GP also benefits from the reconstruction method for the PR trade-off. It reaches 0.995/0.7 with LR mapping (Fig. 3) compared to 0.74/0.61 for the best WGAN-GP generating directly the entire transcriptome (Appendix 14). In light of this comparative study between all the generative models, the heterogeneity and dimensionality of each dataset, the reconstruction method is a proper alternative to generating all the genes.

## Conclusion

This paper presents two diffusion-model frameworks (DDPM and DDIM) and shows that both can be adapted to bulk transcriptomics data generation. To the best of our knowledge, this is among the first demonstrations of diffusion models achieving competitive performance on TCGA and GTEx in this setting, enabled by learning in the reduced L1000 landmark-gene space and reconstructing the full transcriptome.

The extensive comparative validation of these approaches on TCGA and GTEx datasets shows that diffusion models can be competitive with alternative generative models, namely VAEs and WGAN-GP. Regarding predictive accuracy in the reduced L1000 gene space, diffusion-generated data yields performance closest to the baseline obtained using real data. On the positive side, diffusion models are easier to train in terms of stability and convergence; on the negative side, the trained models are approximately one order of magnitude larger than VAEs or GANs. Additionally, we observed that the mapping strategy to the full transcriptome improves all generative pipelines.

In this study, we focused on a controlled benchmark setting (tissue-conditioned generation) to validate realism, diversity, and utility. Extending the framework to clinically targeted endpoints (molecular subtypes, treatment response, survival) will likely require conditioning on additional patient-level covariates and, in practice, adequate sample sizes per label, as well as careful handling of censored outcomes. Beyond clinical endpoints, downstream statistical tasks such as eQTL analysis could also benefit from synthetic expression surrogates [43], provided that genotype–expression dependence is preserved. More broadly, these directions motivate incorporating stronger forms of guidance through covariates, priors, or constraints to steer diffusion trajectories toward clinically and biologically consistent profiles. Finally, another perspective is to exploit interpolation in the learned representation space to better characterize the transcriptomic manifold and support biomarker discovery.

## Figures and Tables

**Fig. 5:**
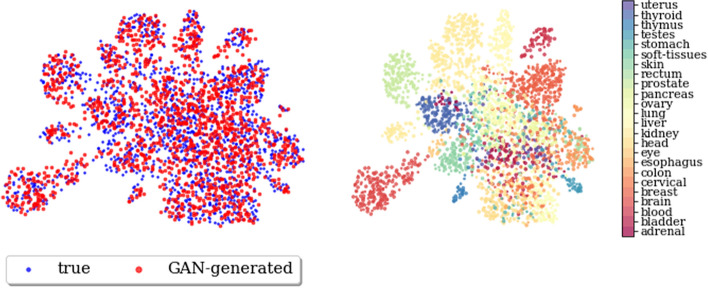
TCGA dataset (L1000): true and GAN-generated data using UMAP visualization. Left: contrasting true and generated samples. Right: contrasting the 24 tissue clusters (in colors)

**Fig. 6:**
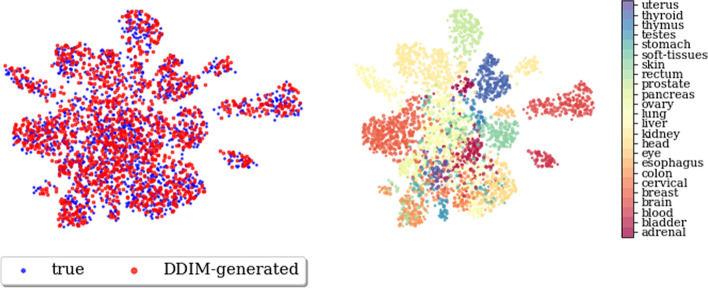
TCGA dataset (L1000): true and DDIM-generated data using UMAP visualization. Left: contrasting true and generated samples. Right: contrasting the 24 tissue clusters (in colors)

**Fig. 7:**
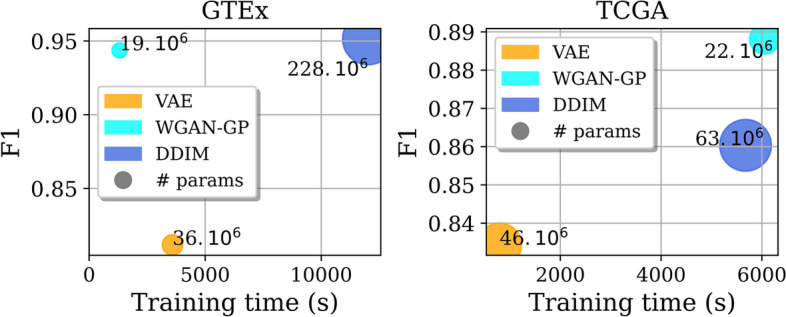
Generative models complexity: number of parameters of the best generative models w.r.t. training time and unsupervised F1 indicator

## Data Availability

Full public code is available at https://forge.ibisc.univ-evry.fr/alacan/rna-diffusion.git. This repository includes preprocessing, training scripts and config files. All the presented experiments consider publicly available data. TCGA data (DOI:10.1038/ng.2764) was retrieved using the RTCGA (https://bioconductor.org/packages/RTCGA) package in R (release date 2016–01-28), while the latest version (v8) of GTEx data (with TPM normalization) was obtained from the open-access portal at: https://gtexportal.org/home/downloads/adult-gtex/bulk_tissue_expression (DOI:10.1038/ng.2653).

## Appendix A Generative baselines

This Section briefly reminds the main generative models used for gene expression so far: Variational Encoders and Generative Adversarial Models. In the following, we reuse the notations from Section Methods.

### A.1 Variational autoencoders

Variational Autoencoders extend the famed auto-encoder architecture as follows. The *encoder* maps input $x \in \mathbb {R}^D$ into the parameters of a distribution noted *Q*(*z*|*x*) in the latent space. The *decoder* samples some $z \in \mathbb {R}^d$ after *Q*(*z*|*x*), and maps *z* onto a sample *x'* in $\mathbb {R}^D$ generated from *P*(*x*|*z*). The VAE learning criterion (Eq. A1) involves two terms; the former one is the reconstruction error (the MSE between *x* and *x'* in the case where *P*(*x*|*z*) is a Gaussian distribution, up to a multiplicative constant). The latter is the Kullback–Leibler divergence between the image distribution *Q*(*z*|*x*) and the target distribution in latent space (usually a Gaussian distribution).

A1 $$\begin{aligned} \underset{z \sim Q(z | x)}{\mathbb {E}}\left[ \log (P(x | z)) - \text {KL}(Q(z | x) || p_{\theta }(z))\right] \end{aligned}$$

### A.2 Generative adversarial networks

A Generative Adversarial Network (GAN) comprises two components, a generator *G* and a discriminator *D* interacting along a zero-sum game. From the true data samples $x \in \mathcal {D}_{t}$, the generator *G* learns a distribution $\mathcal {D}_g$ and uses it to generate synthetic data. The discriminator *D* aims to discriminate between the true and the generated data, while the generator seeks to fool the discriminator. Informally, the generator succeeds when the discriminator fails to distinguish between $\mathcal {D}_{t}$ and $\mathcal {D}_g$. On one hand, GANs capture complex data distributions more easily than VAEs (e.g., when dealing with multimodal distributions). On the other hand, GANs face some challenges in optimizing the min-max learning criterion efficiently. The introduction of Wasserstein GANs (WGANs), particularly the WGAN-GP, addresses these challenges and offers enhanced stability. It mitigates issues arising from non-overlapping distributions by considering their Wasserstein distance via gradient penalties:

A2 $$\begin{aligned} \begin{array}{ll} \underset{G}{\text {min}}\,\underset{D}{\text {max}} & L(D,G) = \mathbb {E}_{x \sim \mathcal {D}_{t}}[D(x|c)] - \mathbb {E}_{z \sim P_z}[D(G(z|c))]\\ & + \lambda \mathbb {E}_{\tilde{x} \sim \mathcal {D}_g}[(\Vert \nabla _{\tilde{x}} D(\tilde{x}) \Vert _2 - 1)^2] \end{array} \end{aligned}$$

The *λ * penalty weight enforces *D* as a 1-Lipschitz function, and $\tilde{x}$ is the linearly interpolated data between true *x* and generated $\hat{x}$ conditioned by context *c*.

## Appendix B Unsupervised indicators definitions

We focused on the panel of indicators investigated by [14] and detailed below.

### B.1 Fréchet Inception Distance (FD)

FD relies on the dimensionality reduction, achieved by mapping a sample *x* onto its activations *y* in some latent space e.g., the latent space of a discriminant neural network. The assumption is that true and generated data should have similar activation functions distributions. In computer vision, the FD is computed using the last pooling layer prior to the output classification of images in the Inception v3 pretrained network [38]. Informally, the true and generated distributions are approximated as Gaussian distributions respectively noted $\mathcal {N}(\mu _t, \Sigma _t)$ and $\mathcal {N}(\mu _g, \Sigma _g)$. FD is the Wasserstein-2 distance between both Gaussian distributions (the lower, the better):

B3 $$\begin{aligned} FD(\mathcal {D}_{t},\mathcal {D}_g) = || \mu _t - \mu _g||^2 + Tr(\Sigma _t + \Sigma _g - 2\sqrt{\Sigma _t.\Sigma _g}) \end{aligned}$$

In computer vision, low FDs appear to be well correlated with higher quality generated images. FD is sensitive to the number of considered samples. We thus adapt the FD measure to transcriptomics by i/ considering all true training data samples; ii/ adjusting the embedding in the low-dimensional space considered to compute FD. Because FD depends on the chosen embedding model and finite-sample estimates, FD values are best interpreted relatively (comparing models under the same protocol).

### B.2 Precision and recall (PR)

PR actually measure the distance between the true and fake distributions, accounting for the intrinsic dimensionality of their support. Formally, precision (respectively recall) is the probability of a sample from $D_g$ (resp. $D_t$) to fall within the support of $D_t$ (resp. $D_g$) ranging in [0, 1]. A high precision suggests that generated data are "close" to true data; a high recall suggests that no true data is "far" from generated data.

Following [39], a hypersphere-based manifold approximation is used to account for the intrinsic dimensionality of the supports. To each (true or generated) sample *x* is associated a ball $B(x, r_k(x))$ centered on *x*, with $r_k(x)$ the distance between *x* and its *k*-th nearest neighbor in $\mathbb {R}^{d}$. The coverage of these balls approximates the manifold *H* containing *x*. The precision is thus defined as the ratio of generated samples falling in $H_t$, and conversely, the recall is the ratio of true samples falling in $H_g$:

B4 $$\begin{aligned} \begin{array}{ll} \text {Precision}(x \in \mathcal {D}_g) = & \left\{ \begin{array}{lll} 1, \text{ iff } x \in H_t \\ 0, \text{ otherwise } \end{array} \right. \\ \text {Recall}(x \in \mathcal {D}_{t}) = & \left\{ \begin{array}{lll} 1, \text{ iff } x \in H_g \\ 0, \text{ otherwise } \end{array} \right. \end{array} \end{aligned}$$

To define whether a point *x* belongs to a manifold *H*, the authors defined the following indicator function *f*(*x*, *H*):

B5 $$ f(x,H) = 1\left[ {\left\| {x - y} \right\|_{2} \le \left\| {y - NN_{k} (y,H)} \right\|\quad for\;at\;least\;one\;y \in H} \right] $$

Each generated sample must fall within at least one hypersphere centered on a real sample for high precision. Each real sample needs to fall within at least one hypersphere centered on a generated sample for a high recall. This improved PR is currently the most adopted approach in deep generative model evaluation, especially in computer vision.

As the curse of dimensionality impacts the relevance of Euclidean distances, we set the number of neighbors *k* to 10 (respectively 50) to enforce stable precision and recall measures on the L1000 genes (respectively all the transcriptome). Like FD, the precision and recall are sensitive to the number of samples, and we consider all true samples in estimating these indicators.

Another performance indicator is the F1-score which is the harmonic mean of the precision and recall measures. Analogously to the FD score, these metrics are computed on the true training set to benefit from a large amount of comparison samples.

### B.3 Adversarial accuracy (AA)

In the context of sensitive data, (Yale et al., 2020) developed the Adversarial Accuracy (AA) metric to assess whether the generated data are neither too far from true data (hindering their utility) nor too close (possibly entailing a breach of privacy). Formally, letting $d_{tt}(i)$ and $d_{tg}(i)$ respectively denote the min distance of the *i*-th true sample to another true (resp. generated) sample, and symmetrically, $d_{gt}(j)$ and $d_{gg}(j)$ denote the min distance of the *j*-th generated sample to another true (resp. generated) sample, it comes:

B6 $$\begin{aligned} AA = \frac{1}{2} \left( \frac{1}{n}\sum _{i=1}^{n} 1(d_{tg}(i)> d_{tt}(i)) + \frac{1}{n}\sum _{i=1}^{n}(d_{gt}(i) > d_{gg}(i))\right) \end{aligned}$$

AA can be understood as the accuracy of a 1-NN classifier discriminating true from generated data. When AA decreases to 0, this suggests that the generated data are too close copies of the true data (the generator overfits the training set). On the contrary, an AA close to 1 indicates that the generated data can easily be discriminated from the true data (the generator underfits the training set). Overall, a generative model with an AA around 0.5 should achieve a good trade-off between accuracy and privacy.

Similarly to precision and recall, AA is based on Euclidean distances in the data space; however, it is not sensitive to the number of nearest neighbors considered according to our experiments. The reported AA thus follows Eq. B6 is computed on a subset of true training samples due to time constraints.

### B.4 Correlation score

While the above indicators globally assess the difference between true and generated data distributions, (Viñas et al., 2021) propose to compare the moments of both distributions. More precisely, the so-called Pearson correlation score is computed from the correlation matrices $M_t$ and $M_g$ associated with true and generated data, as follows:

B7 $$\begin{aligned} \rho (M_t, M_g) = \sum _{i=1}^d \sum {j=i+1}^d \frac{(M_{i,j;t} - M_{i,j;t})}{\sqrt{\sum _{k=1}^d M_{i,k;t}^2 . \sum _{k=1}^d M_{i,k;g}^2 }} \end{aligned}$$

## Appendix C Best hyper-parameters

In the interest of time and resources, we limited the search for the best hyper-parameters to the batch size, learning rate, optimizer, and hidden layers dimensions for the VAE and WGAN-GP over 1000 epochs. As numerical errors can arise with the VAE, it is useful to force the initialization of weights to minimal values ( 8.10–2). As regards the WGAN-GP, the discriminator requires five additional iterations than the generator, and each network needs its own learning rate so that none of them outperforms the other too quickly. The training time is 1 h and 40 min for the best WGAN-GP on TCGA (batch size is 64), and 21 min on GTEx (batch size is 1024).

For generative models, we performed Bayesian optimization rather than random search, with a fixed budget of 100 trials per model: the best configuration maximizes the unsupervised F1 score. For the MLP classifiers, the best hyperparmeters were obtained after maximizing the validation accuracy across 1,000 Bayesian trials (Table 4, Table 5, Table 6, Table 7, Table 8).

**Table 4:** Best hyperparameters for the VAEs trained on landmark genes

	GTEx	TCGA
Batch size	256	2048
Hidden dim. 1 (enc./dec.)	8192	2048
Hidden dim. 2 (enc./dec.)	4096	8192
Hidden dim. 3 (enc./dec.)	4096	256
Hidden dim. 4 (enc./dec.)	2048	4096
Latent dim	128	128
Learning rate	0.0008	0.001
Optimizer	Adam	Adam

**Table 5:** Best hyperparameters for the WGANs-GP trained on landmark genes

	GTEx	TCGA
Batch size	1024	64
Hidden dim. 1 (disc.)	8192	4096
Hidden dim 1 (gen.)	128	256
Hidden dim 2 (disc.)	256	128
Hidden dim 2 (gen.)	1024	1024
Hidden dim 3 (gen.)	4096	8192
Learning rate (disc.)	0.000582	0.000124
Learning rate (gen.)	0.002205	0.000275
Optimizer	RMSprop	RMSprop
Spectral normalization	False	False

**Table 6:** Best hyperparameters for the DDIM trained on landmark genes

	GTEx	TCGA
Beta schedule	quadratic	quadratic
Hidden dim	8192	4096
Dropout	0.1	0.1
Learning rate	0.0005	0.001
Time sinusoidal embedding	False	False

**Table 7:** Best hyperparameters for the baseline MLP trained on landmark genes

	GTEx	TCGA
Batch size	64	2048
Dropout	0.3	0.5
Hidden dim 1	1024	256
Hidden dim 2	1024	16
Learning rate	0.0001	0.004
Optimizer	Adam	Adam

**Table 8:** Best hyperparameters for the baseline MLP trained on all the genes (landmarks and targets)

	GTEx	TCGA
Batch size	1024	64
Dropout	0.5	0.5
Hidden dim 1	2048	1024
Hidden dim 2	8192	1024
Learning rate	0.000516	0.012551
Optimizer	Adam	SGD

## Appendix D UMAP visualization

See Figs. 5 and 6

## Appendix E Additional results

See Table 9, 10, 11, 12, 13, 14 and 15.

**Table 9:** Mean Fréchet distance evolution according to the number of diffusion steps used in inference

Steps	GTEx			TCGA		
	50	100	1000	50	100	1000
DDPM (*η =1*)	**2.0396**	**1.7771**	**1.2114**	**0.2660**	**0.2140**	**0.1730**
DDIM (*η =0*)	4.0588	3.9795	4.0412	0.6572	0.6443	0.5154

Best results for a given dataset and a given number of steps are highlighted in bold. The Fréchet distance decreases with the number of steps

**Table 10:** Mean absolute reconstruction error on test dataset of the best linear regression (LR) and deep learning (MLP) methods

	GTEx (MAE *↓ *)	TCGA (MAE *↓ *)
LR	0.1979 ± 1e-5	0.375± 1e-5
MLP	**0.1543 ± 5e-4**	**0.2696 ± 1e-3**

Best results in bold

**Table 11:** Unsupervised indicators performance on the GTEx dataset w.r.t both reconstruction methods

	LR	MLP
Correlation (*↑ *)	**99.34±0.02**	99.31±0.04
Precision (*↑ *)	**100.0±1e-4**	**100.0±1e-4**
Recall (*↑ *)	97.6±1e-4	**98.55±0.05**
F1 (*↑ *)	98.79±1e-4	**99.27±1e-4**
AA	09.54±0.32	**15.36±0.27**

**Table 12:** Unsupervised indicators performance on the TCGA dataset w.r.t both reconstruction methods. Best results in bold

	LR	MLP
Correlation (*↑ *)	**97.79±0.08**	97.48±0.18
Precision (*↑ *)	**100.0±1e-4**	**100.0±1e-4**
Recall (*↑ *)	**95.4±1e-4**	93.02±2.49
F1 (*↑ *)	**97.65±1e-4**	96.38±1e-4
AA	15.98±0.64	**29.3±2.71**

Best results in bold

**Table 13:** Unsupervised indicators performance on the GTEx dataset w.r.t the best VAE and WGAN-GP trained on both landmark and target genes

	VAE	WGAN-GP
Correlation (*↑ *)	**99.22±0.03**	98.94±0.03
Precision (*↑ *)	**99.93±0.03**	99.73±0.03
Recall (*↑ *)	92.74±0.24	**93.81±0.18**
F1 (*↑ *)	96.2±0.05	**96.68±0.05**
AA	47.89±0.39	**58.47±0.5**

Best results in bold

**Table 14:** Unsupervised indicators performance on the TCGA dataset w.r.t the best VAE and WGAN-GP trained on both landmark and target genes

	VAE	WGAN-GP
Correlation (*↑ *)	**97.08±0.06**	73.12±3.2
Precision (*↑ *)	**100.0±1e-4**	74.57±0.36
Recall (*↑ *)	**79.18±0.36**	61.5±0.11
F1 (*↑ *)	**88.38±1e-4**	67.41±0.17
AA	**46.99±0.22**	82.43±0.5

Best results in bold

**Table 15:** Reverse validation on true tissue types: test accuracy of a MLP trained only on a generated dataset of all landmark and target genes

	GTEx	TCGA
VAE	0.2674±0.1045	0.9449±0.0027
WGAN-GP	0.9916±0.0007	0.937±0.002

All classifiers are trained on the same number of samples and share the same architecture

## Appendix F Models complexity and training time

See Figs. 7

## Footnotes

1 https://bioconductor.org/packages/RTCGA.

2 https://gtexportal.org/home/downloads/adult-gtex.

3 https://forge.ibisc.univ-evry.fr/alacan/rna-diffusion.git.
